# Enhancing Cr (VI) Adsorption of Chestnut Shell Biochar through H_3_PO_4_ Activation and Nickel Doping

**DOI:** 10.3390/molecules29102220

**Published:** 2024-05-09

**Authors:** Wen Hu, Xiaojing Zhang, Ming Chen, Sheikh Tamjidur Rahman, Xin Li, Geming Wang

**Affiliations:** 1 School of Information Engineering, Hubei University of Economics, Wuhan 430205, China; 2 Provincial Key Laboratory of Plasma Chemistry and Advanced Materials, Wuhan Institute of Technology, Wuhan 430073, Chinas.tamjidur@gmail.com (S.T.R.);; 3 Hubei Key Laboratory of Industrial Fume and Dust Pollution Control, Jianghan University, Wuhan 430056, China

**Keywords:** biochar, Cr (VI) removal, activation, doping, liquid purification

## Abstract

A high-efficiency nickel-doped porous biochar (PCNi_3_) has been successfully synthesized from chestnut shell waste via a two-step chemical activation treatment with H_3_PO_4_. The influences of microstructure, surface morphology, elemental composition, surface functional groups, specific surface area, porosity, pore-size distribution, and chemical properties of the surface state on the removal of Cr (VI) from water were thoroughly investigated by using XRD, FESEM, FTIR, Raman, BET, and XPS testing methods, N_2_ adsorption, and XPS testing techniques respectively. The results indicate that the treatment of H_3_PO_4_ activation and nickel doping can effectively improve microstructure characteristics, thus promoting Cr (VI) adsorption capacity. The effects of initial solution pH, solution concentration, time, and temperature on remediation are revealed. The Cr (VI) uptake experiments imply that the adsorption curves of PCNi_3_ fit well with the Freundlich model, the pseudo-second-order kinetic model, and the Elovich model. The adsorption process of PCNi_3_ can be regarded as a spontaneous endothermic reaction limited by diffusion among particles and porosity. The adsorption mechanisms of PCNi_3_ are ion exchange, complexation, electrostatic adsorption, and coprecipitation with the assistance of surface active sites, porosity, Ni^0^ particles, and Ni_7_P_3_. With these advantages, PCNi_3_ reveals an extraordinary Cr (VI) removal capacity and a strong ability to reduce Cr (VI) to Cr (III).

## 1. Introduction

Heavy metals, such as zinc (Zn), lead (Pb), cadmium (Cd), tin (Sn), chromium (Cr), and copper (Cu), are metals with densities greater than 4.5 g·cm^−3^ and originate mainly from human activities, including agriculture, industry, transportation, mining, etc. [1,2]. With the discharge of wastewater, exhaust gas, and solid waste endowed with these heavy metals, surface water resources, the atmosphere, and soil will be directly polluted. With respect to wastewater treatment, various techniques, such as electrolysis, chemical precipitation, ion exchange, membrane separation, and adsorption, have been successfully developed and applied to eliminate heavy metals from wastewater [3,4]. Among these technologies, the adsorption method has outstanding advantages, including a low cost, a simple process, and environmental friendliness, and is considered as efficient as most of the other methods chosen for the removal of heavy metals from wastewater [5,6]. In the past few decades, biochar (mainly from agricultural and forestry wastes) has been considered one of the most promising and environmentally friendly adsorbent materials due to its large specific surface area, unique porous structure, richness in surface functional groups and active sites, and stable physical/chemical properties, as well as having the advantage of “turning wastes into wealth” [6,7]. Biochar, a by-product derived from the pyrolysis of biomass under anaerobic conditions can be used as a substitute for activated carbon that is used in the conventional way to eliminate organic pollutants and heavy metals from wastewater. However, raw biochar usually fails to exhibit satisfactory adsorption behavior owing to its drawbacks, for example, its low specific surface area, the irregular shape of its pore distribution, and its having few adsorption active sites [8,9]. Investigations prior to this one have proven that the mechanism related to the adsorption of heavy metals from wastewater by biochar mainly involves the electrostatic interactions between heavy metal particles or ions and the biochar exterior [10]. The microstructural characteristics determine the activity of complexions and ion exchange of ionizable protons on the surface of biochar as well as the delocalized π–electron interactions on the surface. Therefore, improving the porous structure, modifying the surface functional groups, increasing the surface active sites of biochar, and then optimizing the contact mechanisms between biochar and heavy metals are key to boosting its adsorption performance.

Recently, a variety of modification treatments, such as chemical activation, physical activation, and doping strategies, have been developed and applied to optimize biochar with enhanced heavy metal adsorption capacities. The most commonly used activating reagents are H_3_PO_4_, KOH, NaOH, K_2_CO_3,_ and ZnCl_2_ [11]. As an example, Amali Herath et al. developed modified biochar derived from Douglas fir using a KOH-assisted activation approach, and the highest Langmuir adsorption of Cr (VI) was 124.2 mg·g^−1^ [10]. Nevertheless, the KOH treatment of biochar usually exhibits obvious shortcomings: relatively low carbon production and a hostile impact on the human body as well as the environment. Compared to these methods, H_3_PO_4_ activation treatment can promote the adsorption of heavy metal ions by modulating the acidic functional groups on the surface of biochar [3]. During the activation process, H_3_PO_4_ acts as a dehydration and flame retardant to accelerate the carbonization and aromatization of biochar precursors at low temperatures; meanwhile, H_3_PO_4_ can also inhibit the collapse and shrinkage of the porosity of biochar at high temperatures and ultimately retain the well-developed hierarchical porous structure [12]. Therefore, the application of H_3_PO_4_ as an activator not only promotes bond cleavage reactions but also cross-links to form lamellar linkages, such as phosphate esters and polyphosphate esters, via cyclization and condensation, thus preventing excessive burn-off during carbon activation and achieving the purpose of protecting the internal pore structure [13]. For instance, Zeng et al. fabricated activated eucalyptus char by H_3_PO_4_, the surface area of biochar increasing from 253.25 to 1265.56 m^2^·g^−1^ and the removal rate of Cr (VI) being as high as 99.76% [14]. Yang’s group obtained a novel lignin-based activated porous carbon which resulted in a larger specific surface area of more than 2000 m^2^·g^−1^ by using H_3_PO_4_ activation [15]. In addition, the ability to introduce novel surface functional groups on the surface of biochar as well as magnetic separation with the assistance of magnetic particles in biochar has attracted widespread attention. Chen et al. prepared modified biochar with FeS nanoparticles and chitosan composites, and its equilibrium U (VI) adsorption capacity from aqueous solution could reach as high as 92.45 mg·g^−1^ [16]. Yap et al. reported that the model described by Langmuir was more appropriate for explaining the adsorption of Cd^2+^ and Pb^2+^ by a novel magnetic biochar with a distinguishable behavior, which was successfully synthesized by microwave technology using discarded coconut shells [17]. Recently, Jiang’s group found that the saturated Cr (VI) adsorption capacity of biochar composites obtained by ZnCl_2_ modification combined with FeS loading was as high as 264.03 mg·g^−1^, and its activity of adsorption was in accordance with pseudo-second-order and Redlich–Peterson isotherm models [18].

In this study, a series of chestnut shell-based porous magnetic biochars were successfully prepared by using common agricultural waste chestnut shells as biomass raw materials under the treatment of H_3_PO_4_ activation and using nickel chloride hexahydrate (NiCl_2_∙6H_2_O) as a nickel source. The relationship between the microstructural features of the biochars, e.g., microstructure and morphology, elemental composition, surface functional groups, specific surface area, porous characteristics, and surface chemical state, and the nature of the Cr (VI) elimination from water is explained thoroughly. Based on analyzing the influences of the initial solution pH, the Cr (VI) concentration, the adsorption time, and the reaction temperature on its adsorption performance, the mechanism for the removal of Cr (VI) onto chestnut shell-based modified porous biochar is explored by establishing adsorption kinetic, adsorption isotherm, and adsorption thermodynamic models. Our work lays a certain theoretical and technological foundation for the forthcoming usage of biochar in the elimination of Cr (VI) from water.

## 2. Results and Discussion

### 2.1. The Physicochemical Properties of Adsorbents

**Figure 1:**
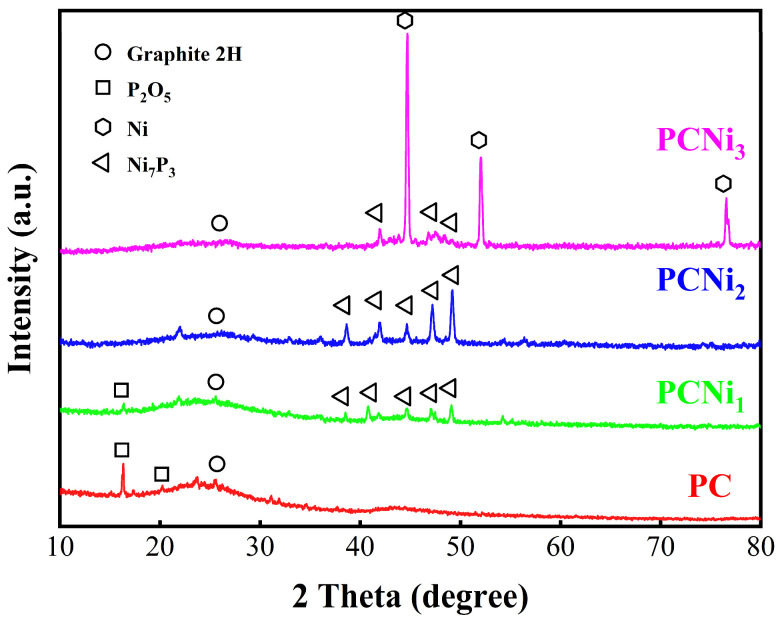
XRD patterns of PC, PCNi_1_, PCNi_2_, and PCNi_3_ can be seen as red, green, blue, and pink line accordingly.

Figure 1 shows the XRD patterns of PC and PCNi. It can be observed that diffraction peaks at 2θ around 26.3°, which are associated with 2H graphitic carbon reflection, exist for all samples [19]. The XRD pattern of PC by H_3_PO_4_ activation demonstrates two typical diffraction peaks at 2θ around 16.4° and 20.2°, which are attributed to the residual P_2_O_5_ (JCPDS card no. 01-0213). Notably, the main diffraction peaks of 38.8°, 41.8°, 44.3°, 47.0°, and 49.5°, corresponding to (321), (400), (330), (420), and (332) diffraction planes of Ni_7_P_3_ (JCPDS card no. 03-1101), can be observed clearly in PCNi by H_3_PO_4_ activation and nickel doping. The reason for the appearance of Ni_7_P_3_ may be that the residual P_2_O_5_ on biochar reacts with NiCl_2_ to form and anchor on the surface of PCNi. The Ni_7_P_3_ has a higher conductivity than that of commercial graphite and can be regarded as an excellent electron donor with a high charge transfer rate, which is expected to accelerate the absorption and reduction of Cr (VI) in PCNi [20]. In addition, as the nickel doping increases, the diffraction peaks of PCNi_3_ at 44.5°, 51.8°, and 76.4° can be ascribed to (111), (200), and (200) crystal planes of Ni^0^ (JCPDS card no. 04-0850), respectively, which indicates that the ions of Ni are partially transferred to a condition of a metallic Ni^0^ [21]. Owing to the presence of Ni^0^ and Ni_7_P_3_, the as-prepared biochar possesses the unique advantage of being magnetically recyclable after adsorption. In summary, our XRD results confirm that the nickel ions are successfully loaded into the biochar in the form of metallic Ni and Ni_7_P_3_ through H_3_PO_4_ activation and nickel doping.

**Figure 2:**
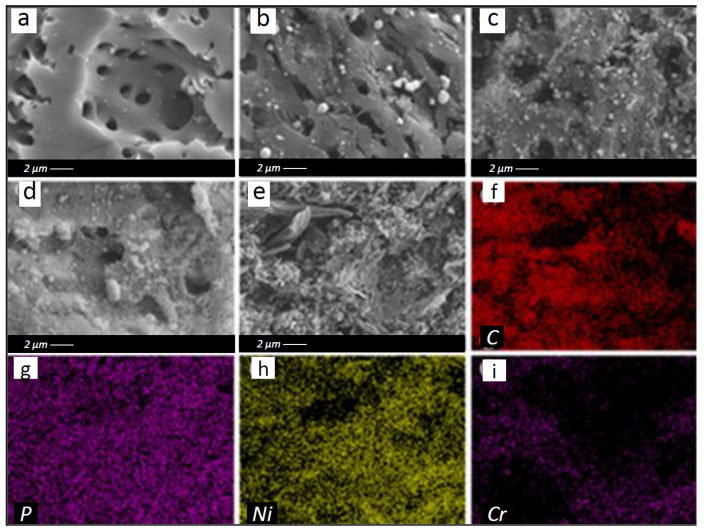
FESEM images of (**a**) PC, (**b**) PCNi_1_, (**c**) PCNi_2_, (**d**) PCNi_3_, and (**e**) PCNi-Cr and the EDS spectra of (**f**) the C element distribution on PCNi_3_, (**g**) the O element distribution on PCNi_3_, (**h**) the Ni element distribution on PCNi_3_, and (**i**) the Cr element distribution on PCNi-Cr.

The superficial or surface morphology and fundamental components of the elemental properties of PC, PCNi, and PCNi-Cr were investigated by FESEM and the equipped EDS mapping methods. After H_3_PO_4_ activation, the PC presented a three-dimensional honeycomb-like structure and an irregular porous structure consisting of many well-developed porosities (Figure 2a), which could provide it with a remarkably huge specific surface area and an abundance of surface active sites, thereby improving the Cr (VI) adsorption performance remarkably. This indicates that H_3_PO_4_ activation has a major consequence for the microstructure of chestnut shell-based biochar. After the treatment of Ni doping, the surface of PCNi gradually showed a trend of roughness, and numerous particles as well as cotton-like substances were found on the surface pores of PCNi (Figure 2a–d). Moreover, as the nickel content increased, the cotton-like substances became more apparent. In PCNi_3_, we could observe that its entire surface was covered by a layer of reticulate cotton-like material (Figure 2d). Combining these results with the XRD results, it was speculated that these particles and cotton-like substances should be the metallic-state Ni^0^ and Ni_7_P_3_ formed during the annealing process. Although this Ni-containing matter can block some surface pores of biochar and deteriorate its specific surface area and porous structure, the existence of Ni^0^ and Ni_7_P_3_ is believed to be beneficial for attracting Cr (VI) ions through electrostatic interaction, thus promoting their anchoring on the surface of the adsorbent. However, the network cotton-like structure basically disappeared and some flocculent condensates were observed on the top layer of PCNi-Cr after Cr (VI) adsorption (Figure 2e). This may be ascribed to the collaboration between Cr (VI) ions and Ni^0^, Ni_7_P_3_, and the related functional groups on its surface. Figure 2f–i show the distribution of C, P, and Ni elements in PCNi_3_ and Cr elements in biochar post the adsorption action of Cr (VI). The elemental compositions of the developed biochars are listed in Table S1. It can be seen that C, P, and Ni are uniformly distributed on the surface of PCNi_3_. In addition, the existence of Cr on the top layer of PCNi-Cr confirms that PCNi_3_ can effectively adsorb Cr (VI) ions from water. The FESEM and EDS results prove that the H_3_PO_4_ activation and Ni doping approach can effectively promote the formation of a porous structure in chestnut shell-based biochar, which may possibly have a more efficient outcome in terms of the adsorption of Cr (VI) from water.

**Figure 3:**
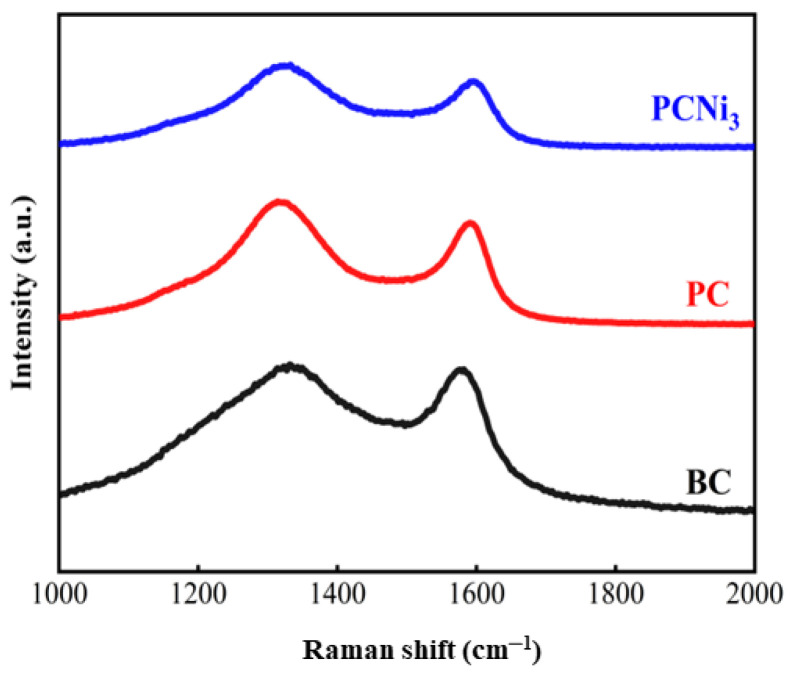
Raman spectra of BC, PC, and PCNi_3_.

In order to investigate the graphitization state of the prepared biochar, Raman spectra of BC (biochar), PC, and PCNi_3_ were obtained and are shown in Figure 3. The obvious characteristic peaks can be observed at ~1322 and ~1592 cm^−1^ in the spectra of all biochar samples, corresponding to the D and G peaks for carbon materials [22]. The D peak represents the sp^3^ hybridization of carbon atoms, disordered vibration of carbon atoms, and defects of carbon atoms, while the G peak can be ascribed to the in-plane telescopic vibration of sp^2^ hybridization of carbon atoms [23,24]. It can be assumed that the D peak represents the relative content of defective carbon and the G peak reflects the relative content of graphitized carbon in the biochar [24]. Therefore, the ratios between D and G peaks (I_d_/I_g_), which reflect the acuteness of defects and disorders in the carbon structure of biochar, can be calculated to be 1.23, 1.40, and 1.46, respectively. The higher values for I_d_/I_g_ indicate that the as-prepared PCNi_3_ treated by H_3_PO_4_ activation and nickel doping had more defects, an enriched pore structure, and a higher degree of disorder than BC and PC, which could endow PCNi_3_ with abundant active sites conducive to the adsorption of Cr (VI) [25].

**Figure 4:**
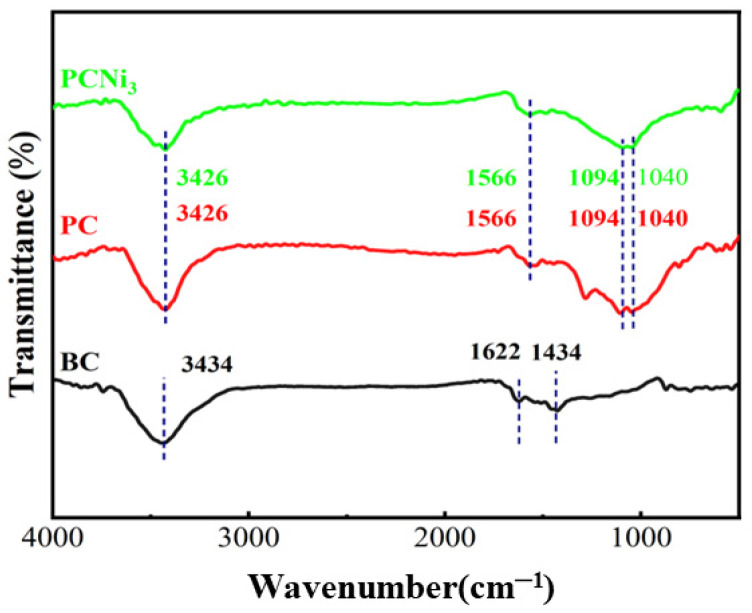
FTIR spectra of BC, PC, and PCNi_3_.

The surface chemical functional groups of the BC, PC, and PCNi_3_ were characterized using FTIR spectra, and the results are displayed in Figure 4. The distinct peak characteristics at 3434, 1622, and 1434 cm^−1^ for BC are correlated with C-O bonds, and some stretching can be noticed due to the aromatic rings of C=O, C=C groups, which can be seen in the structure of lignin as well as in O-H bonds [21]. After H_3_PO_4_ activation and nickel doping, the absorption peaks of PC and PCNi_3_ were shifted and some new peaks appeared at 3426, 1566, 1094, and 1040 cm^−1^, which could have been an effect of -OH groups, aromatic C=C structural vibration, phenolic hydroxyl O-H deformation, C-O stretching vibration, O-H distortion of alcohol or ether groups, C-O elongating vibrations, etc. [26,27]. The shifts in the peaks representing the character and appearance of functional groups on the top layer imply that the enhancement of oxygenated reactive groups in PC and PCNi_3_ provided abundant surface active sites for the adsorption of Cr (VI) from water and thus enhanced the potential for binding between biochar and Cr (VI) [28].

**Figure 5:**
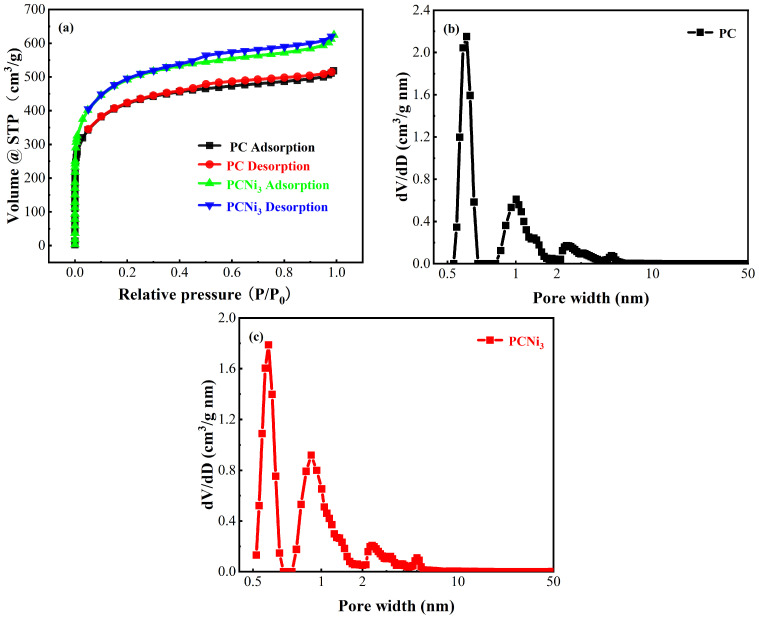
(**a**) The N_2_ adsorption and desorption isotherms of PC and PCNi_3_ and the pore-size distribution plots for (**b**) PC and (**c**) PCNi_3_.

Figure 5 shows the adsorption/desorption as well as the pore-size distribution plots for PC and PCNi_3_. As Figure 5a shows, the curves of all the samples show typical type IV features in the IUPAC classification and H4 hysteresis loop characteristics. The curves rise in the low-pressure part and present an upward, slightly convex shape, which is attributed to the condensation of N_2_ molecules with the gas molecules on the pre-adsorbed layer, indicating the existence of a porous hierarchical structure in the as-prepared biochar [29]. Furthermore, all samples exhibit a rapid rise in the isotherms at the low-pressure end and hysteresis loop presence, implying the existence of more micropores [30]. From the pore-size distribution plots (Figure 5b), the pore-size distribution of PC is mainly concentrated in the range of 0.5~3.0 nm. However, the pore-size distribution of PCNi_3_ does not change much compared to PC (Figure 5c), implying that Ni_7_P_3_ formed during nickel loading does not cause large-scale pore clogging of biochar. According to the parameters regarding the pore structures of PC and PCNi_3_ listed in Table S2, compared to PC, the specific surface area of PCNi_3_ increases from 1521.55 to 1775.94 m^2^·g^−1^, the average pore size increases from 2.11 nm to 2.18 nm, and the total pore volume surges from 0.80 to 0.97 cm^3^·g^−1^. Notably, the microporous area of PCNi_3_ is 1235.85 m^2^·g^−1^, which accounts for 69.59%, which is ascribed to the fact that the gas molecules released during the reduction of doped nickel by carbon produce more micropores as they escape from the surface of biochar. All in all, the PCNi_3_ has a reasonable pore structure and pore-size distribution which is a combination of a high specific surface region and a remarkable microporous structure, such that PCNi_3_ contains more active sites, which promotes attraction between PCNi_3_ and Cr (VI), making it an ideal adsorbent.

**Figure 6:**
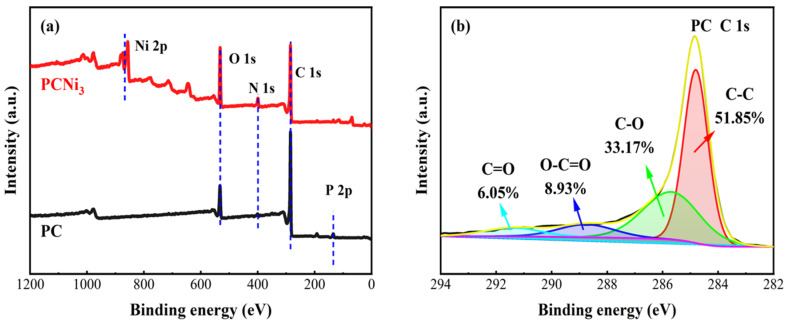

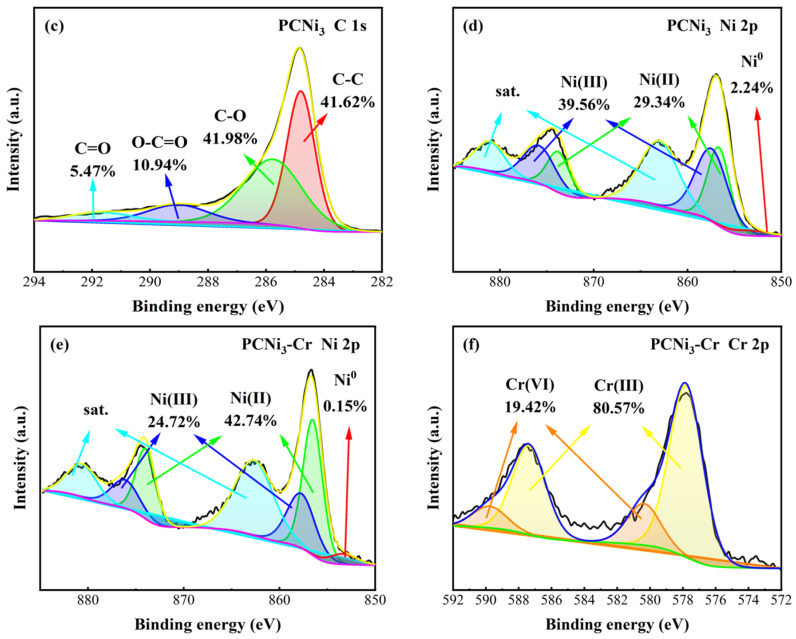
XPS spectra of biochar: (**a**) XPS full spectra of PC and PCNi_3_, (**b**,**c**) C 1s of PC and PCNi_3_, (**d**,**e**) Ni 2p of PCNi_3_ and PCNi_3_-Cr, and (**f**) Cr 2p of PCNi_3_-Cr.

The surface chemical compositions and elemental valence states of PC, PCNi_3,_ and PCNi_3_-Cr were revealed by XPS analysis (Figure 6). Figure 6a represents the high-resolution XPS full spectra of PC and PCNi_3_. The characteristic peaks located at ~284.9 eV, ~400.4 eV, ~532.8 eV, and ~134.4 eV for the two samples correspond to C 1s, N 1s, O 1s, and P 2p, respectively, which can be attributed to by-products produced by H_3_PO_4_ activation and the self-contained nitrogen element in the biomass itself [31]. Compared with PC, the peak that represents Ni 2p (~856.9 eV) appeared in the XPS pattern of PCNi_3_, reconfirming the successful loading of nickel onto the surface of the biochar. Figure 6b,c exhibit the high-resolution XPS C 1s spectra of PC and PCNi_3_. The clear C 1s spectra which can be fitted to four peaks centered on the ~284.8 eV, ~286.0 eV, ~288.5 eV, and ~289 eV binding energies, which correspond to the presence of graphite/aliphatic C-C bonds, C-O bonds in alcohol/ether groups, O-C=O groups, and C=O bonds in carbonyl groups, respectively [32]. These surface functional groups which contain oxygen play a key role in two major activities: the ion exchange reaction and the complexation process between biochar and Cr (VI). Figure 6d,e show the high-resolution XPS Ni 2p spectra of PCNi_3_ and PCNi_3_-Cr. The Ni 2p of samples located at ~853.35 eV, ~856.66 eV/~873.77 eV, and ~857.42 eV/~875.83 eV correspond to Ni^0^, Ni (II), and Ni (III), respectively [33]. The presence of Ni^0^ particles is consistent with our previous XRD results. Please note that after absorbing Cr (VI), Ni^0^ particles on the surface of PCNi_3_-Cr basically disappear, while the relative amount of Ni (II) rises from 29.34% to 42.74%. This clearly indicates that during the adsorption reaction, Ni^0^ particles participate in the redox process of Cr (VI), which is oxidized to Ni (II) and then gradually consumed. After the absorption process (Figure 6f), the signal produced from Cr 2p of PCNi_3_-Cr can possibly be interpreted as binding energies of ~577.81 eV/~587.37 eV and ~580.38 eV/~589.77 eV, which are attributed to Cr (III) and Cr (VI), respectively [34]. The relative contents of Cr (III) and Cr (VI) were calculated to be 80.57% and 19.43%, respectively, as determined by the peak fitting of Cr 2p, which proves once again that part of the Cr (VI) can be converted to Cr (III) through the reduction process and endowed on the surface of biochar. The above XPS results fully imply that Cr (VI) ions are adsorbed onto the pore-containing surface of the biochar; meanwhile, the chemical interaction between Cr (VI) and the oxygen-containing functional groups (e.g., C-O, O-C=O, C=O, etc.) as well as Ni^0^ particles on the biochar top layer lead to part of Cr (VI) being converted into Cr (III).

### 2.2. The Magnetic Properties of Adsorbents

**Figure 7:**
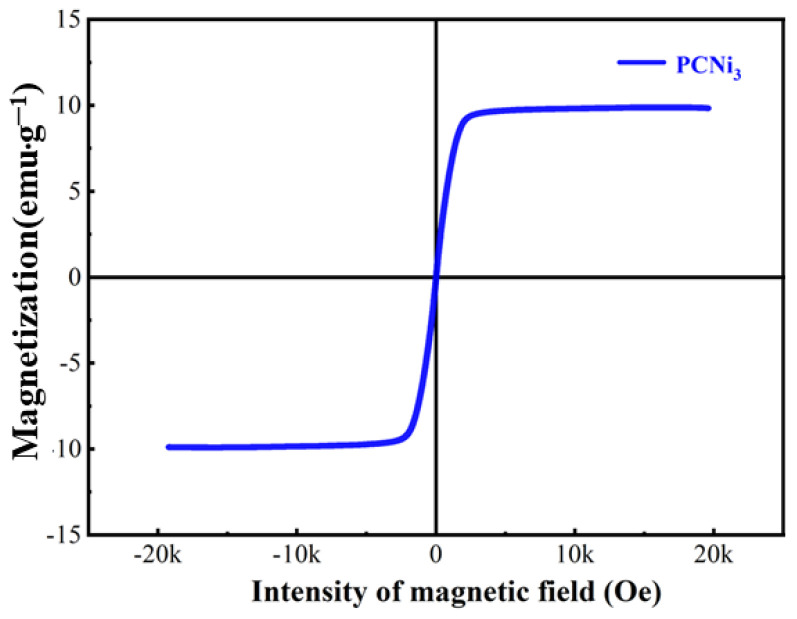
Magnetic hysteresis loop of PCNi_3_.

The magnetic field-dependent magnetization (M-H) illustrated in Figure 7 represents loops in a range of ±2 T at room temperature of PCNi_3_. The remnant and spontaneous magnetization are 0.15 and 9.88 emu·g^−1^, respectively. When the external magnetic field is removed, PCNi_3_ has almost no remnant magnetization and the area of hysteresis loop closure tends to zero, revealing superparamagnetic characteristics at room temperature. This indicates that PCNi_3_ possesses solid–liquid separation and recycling performance under magnetic field application.

### 2.3. Effect of Initial Solution pH on the Adsorption

**Figure 8:**
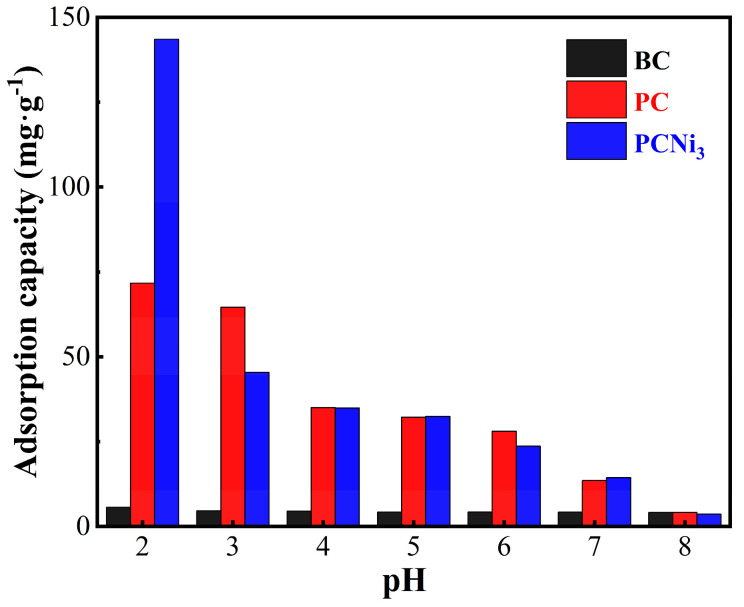
Effects of the initial solution pH on the adsorption of Cr (VI) onto BC, PC, and PCNi (basic conditions: C_e_ = 50 mg L^−1^; V = 100 mL; t_contact_ = 1440 min; T = 298 K).

The initial pH of a solution is an important factor which affects the Cr (VI) adsorption capacity of biochar. The solution pH not only determines chromium’s state in the reaction solution but also can affect the surface functional group distribution states of biochar [35]. As displayed in Figure 8, a higher initial solution pH leads to a weaker Cr (VI) adsorption capacity of biochar. This is because in an acidic environment (pH less than 6.0), the biochar surface can be charged positively by combining with more H^+^, which in turn promotes the adsorption performance of negatively charged Cr (VI) in the form of anions which contain oxygen [36]; when the pH rises, the OH^−^ which become more numerous as a consequence will compete for active sites with anionic groups containing Cr (VI), thereby inhibiting the removal of Cr (VI) [35]. The best removal performance with respect to Cr (VI) was observed in PCNi_3_ (up to 143.51 mg·g^−1^) when the solution pH was 2.0. Furthermore, PCNi_3_ is more influenced by the initial solution pH compared to PC. One possible explanation is that biochar treated by H_3_PO_4_ activation mainly relies on its remarkable porous microstructure and surface functional units for adsorbing Cr (VI), whereas the nickel-doped modified biochar also suffers from the mutual attraction and redox effect between Ni^0^ particles or nickel-based compounds on the surface and Cr (VI), and its performance is much more sensitive to the change in H^+^ in solution. The subsequent batch experiments in this paper were carried out under an acidic environment in which the initial solution pH was 2.0.

### 2.4. Effect of Initial Solution Concentration on Adsorption

**Figure 9:**
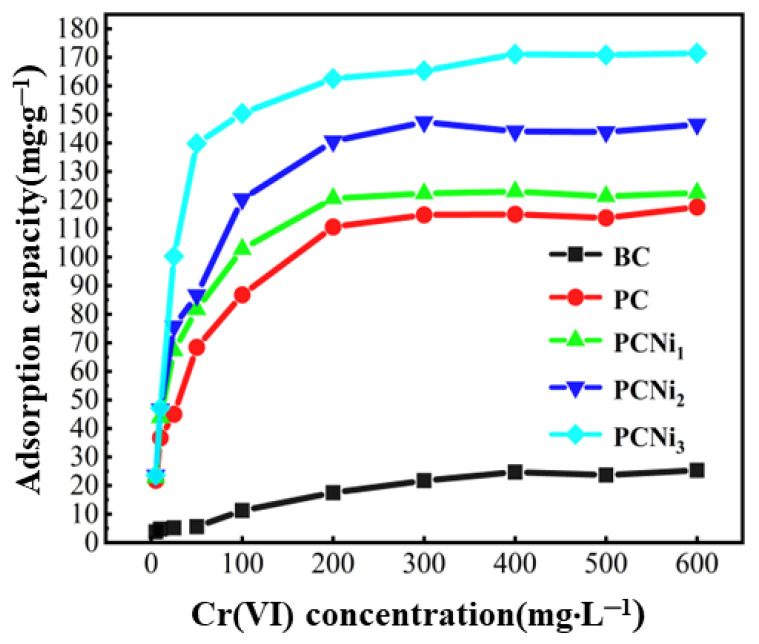
Effect of primary solution concentration on Cr (VI) adsorption (basic conditions: pH = 2.0; V = 100 mL; t_contact_ = 1440 min; T = 298 K).

The primary concentration of a solution is also one of the important parameters affecting the effectiveness of the adsorption process. Figure 9 illustrates the performance of BC, PC, and PCNi in removing Cr (VI) from water under changed initial Cr (VI) concentrations (5~600 mg·L^−1^). With the increment in the initial concentration from 5 to 600 mg·L^−1^, the adsorption of Cr (VI) by modified biochar Cr (VI) firstly increases sharply and then slows down until reaching the adsorption equilibrium. This phenomenon is a result of the fact that when Cr (VI) ions increase in the solution, the limited active regions of the top layer of the biochar are progressively exhausted to reach adsorption saturation [37]. Notably, the adsorption performance of PC and PCNi is much higher than that of BC, indicating that the treatments of H_3_PO_4_ activation and nickel doping have significant modification effects on biochar. In addition, the high content of nickel doping is favorable to the enhancement of the adsorption efficiency of PCNi, which is the result of change in the surface functional groups caused by nickel doping, the strong attraction of surface nickel to Cr (VI), and the high charge mobility rate of Ni_7_P_3_. When the initial solution concentration of Cr (VI) is 600 mg·L^−1^, the PCNi_3_ sample has the highest adsorption efficiency, reaching up to 171.43 mg·g^−1^. The variation in the adsorption efficiency of biochar in the initial solution concentrations of Cr (VI) reflects the results of the competition between the amount of Cr (VI) present in the solution and the number of functional groups and active sites on the external layer of the biochar.

**Figure 10:**
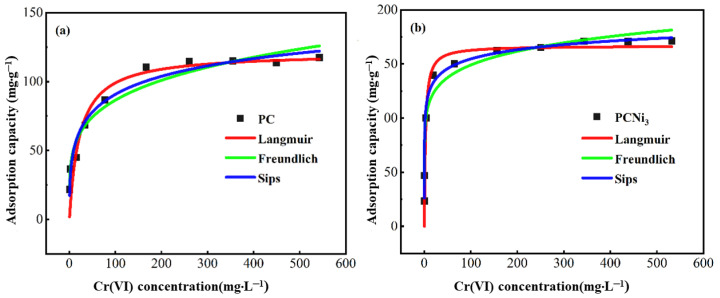
Adsorption isothermal model fitting curves for Cr (VI) using (**a**) PC and (**b**) PCNi_3_ (basic conditions: pH = 2.0; V = 100 mL; t_contact_ = 1440 min; T = 298 K).

Adsorption isotherms were obtained to study the interaction of PC and PCNi_3_ with Cr (VI) at the time of adsorption, and three isothermal adsorption models, namely, the Langmuir, Freundlich, and Sips models, were used to fit the results of the experiment (T = 298 K). The experimental data and simulated plots are shown in Figure 10 and Table 1. The Cr (VI) absorption capacities of PC and PCNi_3_ are 117.52 and 171.43 mg·g^−1^, respectively, confirming that the elimination of Cr (VI) via adsorption onto porous biochar after the treatments of H_3_PO_4_ activation and nickel doping was dramatically enhanced. Meanwhile, the R^2^ values representing the correlation coefficient values of the Langmuir, Freundlich, and Sips adsorption isotherm models for the samples are all greater than or close to 0.9, suggesting that all three models can be used to describe the Cr (VI) adsorption behavior of biochar, which is in agreement with the previous investigations on sludge-based and fine-leaved centipede grass biochar [38,39]. Clearly, the theoretical saturated adsorption capacities in the Langmuir models for PC and PCNi_3_ (121.09 and 166.89 mg·g^−1^) coincide with the experimental outcomes of the samples (117.52 and 171.43 mg·g^−1^). Moreover, the values of the parameter 1/*n_F_* in the Freundlich model for PC and PCNi_3_ are less than 0.5. In general, a smaller 1/*n_F_* value represents easier adsorption action. The fact that it is easily adsorbed when 0.1 < 1/*n_F_* < 0.5 and adsorbed with difficulty when 1/*n_F_* > 2 confirms that the adsorption of Cr (VI) by PC and PCNi_3_ can easily be carried out [40,41]. The gains in *m* mentioned in the Sips model represent the inhomogeneity of the adsorbent. When the value of *m* is closer to 1, the surface of the adsorbent is more homogeneous, and the Sips model will be transformed into the Langmuir model, whereas when the deviation of the value of *m* is 1, it comes closer to the Freundlich model [42,43]. The values of *m* for PC and PCNi_3_ are greater in the deviation from 1, and the R^2^ value in the Langmuir model is also lower than that in the Freundlich model; this represents the heterogeneous adsorption sites on the surface of the biochar, and the Freundlich model fits the actual experimental data more effectively. To sum up, the surface assimilation or adsorption of Cr (VI) by PC and PCNi_3_ is both physical and chemical, and the adsorption process is easy, multilayered, and non-uniform.

### 2.5. Time Effect on Adsorption

**Figure 11:**
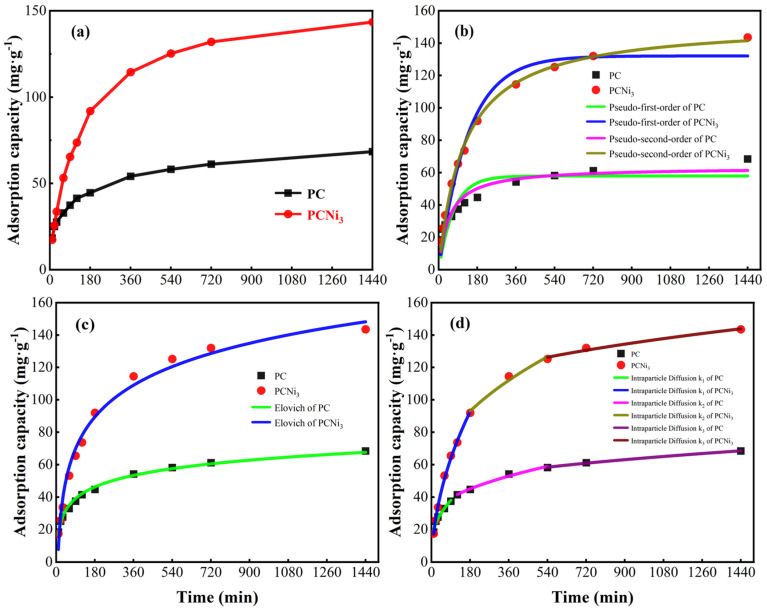
(**a**) Effect of reaction time on Cr (VI) adsorption capacity of PC and PCNi_3_; (**b**) pseudo-first-order kinetic and pseudo-second-order kinetic model plots for Cr (VI) adsorption; (**c**) Elovich kinetic model plot for Cr (VI) adsorption; and (**d**) intra-particle diffusion model plot for Cr (VI) adsorption (basic conditions: pH = 2.0; C_e_ = 50 mg L^−1^; V = 100 mL; T = 298 K).

To study the adsorption of Cr (VI) onto biochar, the adsorption capacities vs. reaction time curves for PC and PCNi_3_ were determined (Figure 11a). Evidently, the adsorption efficiency of biochar with respect to Cr (VI) increases rapidly with the increase in the reaction time in the initial stage of 180 min. The adsorption increases only gradually until the reaction reaches the equilibrium point. Taking PCNi_3_ as an example, its unique porous microstructure, including rich active sites and surface functional groups, can capture Cr (VI) in the preliminary stage of the adsorption process easily. The Cr (VI) ions diffuse rapidly on the surface of the biochar and gradually occupy those active sites [44]. With the increase in reaction time, pores on the surface of PCNi_3_ will be filled, the active sites occupied, and the surface functional groups gradually consumed. Consequently, when the equilibrium is reached, the adsorption efficiency tends to stabilize. At this time, the adsorption capacity of PCNi_3_ reaches 143.51 mg·g^−1^ at its highest.

To deeply analyze the chemistry behind the adsorption of Cr (VI) removal by PC and PCNi_3_, intra-particle diffusion, Elovich, pseudo-first-order, and pseudo-second-order models were applied to fit our experimental data, and Figure 11b–d and Table 2 and Table 3 represent the outcomes and parameters, respectively. The depicted pseudo-first-order kinetic model describes the diffusion steps that control the adsorption; on the other hand, when the adsorption rate is controlled by chemisorption, it is known as the pseudo-second-order kinetic model, which is based on the assumption that the process can be described as electron sharing or electron transfer between the adsorbent and the adsorbate [44]. The chemisorption by covalent sharing or exchange of electrons between the adsorbate and bi-polar functional groups on the surface of the adsorbent is the rate-limiting step [45]. The pseudo-second-order model can better explain the adsorption activity of Cr (VI) by these two biochars, which is confirmed by Table 2, which contains the R^2^ values, i.e., the chemisorption mechanism controls the elimination of Cr (VI) [46]. However, for both PC and PCNi_3_, the theoretical adsorption capacity fitted by the pseudo-second-order model still shows a certain discrepancy with our experimental data, which implies that the adsorption kinetics cannot be explained properly by the pseudo-second-order model. It is noteworthy that the experimental results were found to fit well with the Elovich model as specified by an R^2^ value higher than 0.98. The Elovich model is a combination of a reaction rate and a diffusion factor, which is regarded as a non-homogeneous diffusion process [47]. The validation of the Elovich model indicates that the energy of the biochar surface is inhomogeneous, further confirming the chemisorption (chemical reaction)-dominated nature. Our adsorption kinetics results demonstrate that Cr (VI) ions in water diffuse into the surface pores of PC and PCNi_3_ and react with surface oxygen-containing functional groups (e.g., C-O, O-C=O, C=O, etc.) and Ni^0^ particles via sharing or exchanging electrons, resulting in the conversion of part of Cr (VI) into Cr (III). In addition, the Ni_7_P_3_ on the surface of PCNi_3_ acts as an electron donor with high charge mobility, thus further promoting the adsorption process [48]. Meanwhile, the Elovich model fitting parameter (*α*) values for both PC and PCNi_3_ are much larger than the respective *β* values, which are considered to be related to the initial adsorption/desorption rates (the *α* value represents the adsorption rate, and the *β* value reflects the coverage of active sites). In particular, the *β* values are much lower than the *α* values, which implies that the desorption rate of Cr (VI) from biochar is not as high as the adsorption rate, suggesting that the removal of Cr (VI) in the initial stage is very rapid and that the chance of Cr (VI) escape from biochar is relatively very small, which endows biochar with an excellent adsorption performance [47,49]. The adsorption behavior is a process of non-homogeneous diffusion of Cr (VI) onto PC and PCNi_3_.

The hierarchical control step is also considered an important factor in the process of adsorption of biochar. Figure 11d and Table 3 display the poly-linear connection between rate constants and fitting properties derived from fitted curves of the intra-particle diffusion model. The adsorption mechanism of PC and PCNi_3_ can be controlled by three steps via porous structure: membrane diffusion, boundary layer diffusion, and intra-particle diffusion, which correspond to the transfer of solute from the aqueous medium to the surface of the adsorbent in the form of membrane diffusion, the diffusion of solute in the pore spaces of the adsorbent, and the adsorption of ions on the inner surface of the adsorbent, respectively [24]. Among them, the first high slope of the linear curve represents surface adsorption, namely, the membrane diffusion stage. In this step, Cr (VI) ions diffuse to the surface of biochar through the solution. The second linear curve is steeply sloping, which confirms that the diffusion processes of Cr (VI) on PC and PCNi_3_ are ruled by boundary layer diffusion. The third curve, which is relatively flat (defined as intra-particle diffusion), denotes the steady adsorption of Cr (VI) by PC and PCNi_3_ into micro-/meso-/macropores. The value of the *k*_3_ parameter (intra-particle diffusion) is much lower than the *k*_1_ (membrane diffusion) and *k*_2_ (boundary layer diffusion) values, indicating that the rate of adsorption is mainly controlled by intra-particle diffusion during the adsorption process [50]. Moreover, the third linear curve intersects the origin point in the graph, which indicates that the intra-particle dispersion and absorbency together influence the rate of adsorption of Cr (VI) by PC and PCNi_3_.

### 2.6. Thermodynamic Investigation

**Figure 12:**
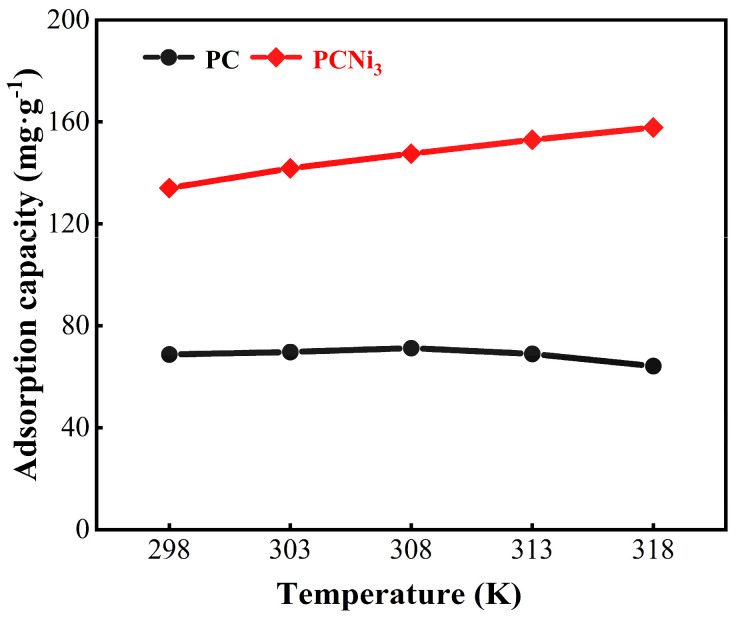
Effect of temperature on the Cr (VI) adsorption capacity of PC and PCNi_3_ (basic conditions: pH = 2.0; C_e_ = 50 mg L^−1^; V = 100 mL; t_contact_ = 1440 min).

To study the spontaneity of adsorption reactions, the adsorption behaviors of the biochar with respect to Cr (VI) were analyzed at various temperatures through adsorption thermodynamics. As demonstrated in Figure 12, the Cr (VI) removal performances of PC and PCNi_3_ at different temperatures, i.e., 298 K, 303 K, 308 K, 313 K, and 318 K, were tested. The Cr (VI) adsorption capacity of PC increases and then falls with the increment in temperature. One possible reason is that when the adsorption equilibrium is reached, the continuously rising temperature increases the entropy of the environmental system and the tendency of active Cr (VI) on the surface of PC increases until it acquires enough energy to be desorbed [38]. Compared with PC, the efficiency of adsorption of Cr (VI) by PCNi_3_ develops with the increase in temperature because the adsorption procedure does not reach equilibrium, and the increase in temperature prompts Cr (VI) ions to move more vigorously in solution, which raises the chances of contact between them and the active sites of biochar [38]. Meanwhile, the rising temperature can also effectively promote the diffusion of particles, which is conducive to the adsorption performance of biochar.

Table 4 lists the related thermodynamic parameters of Cr (VI) adsorption by PC and PCNi_3_. One can see that the Gibbs free energies (ΔG^0^, KJ mol^−1^) of PC and PCNi_3_ are negative at different temperatures, indicating that the adsorption of Cr (VI) by PC and PCNi_3_ is spontaneous and thermodynamically favorable. The ΔG^0^ value of PCNi_3_ decreases with increasing temperature, which implies that a higher temperature leads to a higher driving force of adsorption and is more favorable for adsorption reactions taking place [51]. Positive and negative values of enthalpy (ΔH^0^, KJ mol^−1^) can indicate whether an adsorption mechanism is a heat-absorbing or exothermic reaction. The negative value of ΔH^0^ for PC suggests that the mechanism of adsorption of Cr (VI) by PC is an exothermic reaction. In contrast, the ΔH^0^ value of PCNi_3_ is greater than zero, which indicates that the adsorption behavior of Cr (VI) by PCNi_3_ is an adsorptive reaction, confirming our experimental phenomenon where the adsorption capacity of PCNi_3_ increases with the increase in temperature. The entropy (ΔS^0^, KJ mol^−1^ K^−1^) represents the entropy change in the reaction, and its positive value indicates an increase in disorder at the solid–liquid border. The positive value of ΔS^0^ for PCNi_3_ is due to the fact that the adsorption of Cr (VI) by PCNi_3_ is accompanied by a chemical reaction which produces by-products resulting in the enhancement of entropy in the liquid-phase system. Similarly, Qu et al. investigated the adsorption thermodynamics of corn straw-based porous biochar, and the results showed that both the values of ΔH^0^ and ΔS^0^ were positive [52]. Overall, the adsorption of Cr (VI) by PCNi_3_ can be regarded as a voluntary endothermic reaction. An increase in temperature will improve the diffusion of Cr (VI) ions, which is conducive to the activation of active sites on the surface of the biochar and enhances the affinity between Cr (VI) and the biochar.

### 2.7. Removal Mechanism

**Figure 13:**
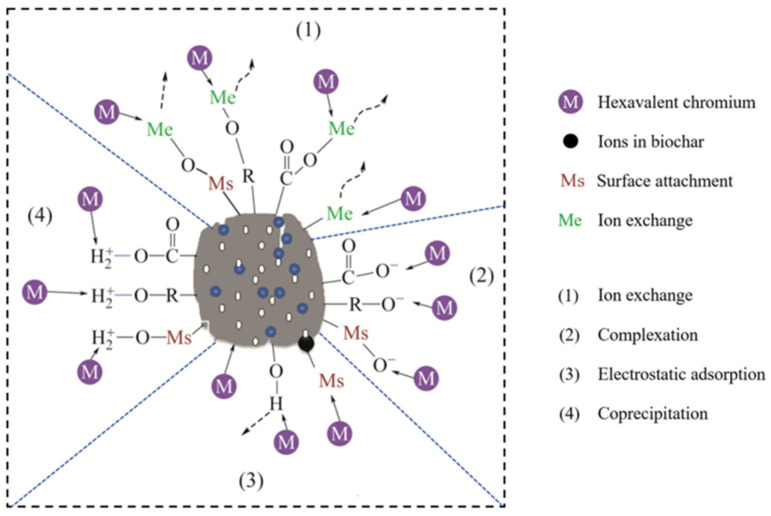
Removal process of Cr (VI) by PCNi_3_.

A process of elimination of Cr (VI) by PCNi_3_ has been discussed and is illustrated in Figure 13. As discussed before, the adsorption behavior of PCNi_3_ is dominated by chemical adsorption and accompanied by physical adsorption. Firstly, the activation of H_3_PO_4_ can widen pore channels and increase the specific surface area of biochar to accelerate the physical adsorption process. The activation mechanism involves H_3_PO_4_ acting as a dehydrator and flame retardant to promote carbonization and aromatization of the biochar at low temperatures. It will be converted into different condensed forms to dissolve into biochar and inhibit material from collapsing and shrinking at high temperatures during heating, thus forming a well-developed pore microstructure [12]. The contact between Cr (VI) ions and PCNi_3_ occurs via (3) electrostatic adsorption with the assistance of surface active sites and porosity, as displayed in Figure 13. The relevant expressions are shown in Equations (1) and (2):

(1) $${2H}_{3}PO_{4}\overset{>213\;{}^{\circ}C}{\to }H_{4}P_{2}O_{7}+H_{2}O$$

(2) $$H_{4}P_{2}O_{7}\overset{>300\;{}^{\circ}C}{\to }2HPO_{3}+H_{2}O$$

Secondly, the appearance of Ni^0^ after nickel doping can be attributed to the reduction of nickel by carbon. The gas released during this process leads to the creation of abundant micropores in the biochar outer layer [48]. The related reactions are illustrated in Equations (3)–(5).

(3) $$5nNiCl_{2}+{\left(C_{6}H_{10}O_{5}\right)}_{n}\to 5nNiO+10nHCl+6n\;\;\;\;\;\;\;\;$$

(4) $$NiO+C\to C^{\prime }+Ni+CO$$

(5) $$CO+NiO\to Ni+CO_{2}$$

Then, in an acidic environment, the surface active groups of biochar and Ni^0^ undergo reduction and complexation reactions with Cr (VI) [53]. These chemisorption processes can be described in Equations (6)–(12).

(6) $$3Ni+{Cr}_{2}O_{7}^{2-}+14H^{+}\to 3{Ni}^{2+}+2{Cr}^{3+}+7H_{2}O$$

(7) $$R-COO^{-}+{Cr}^{6+}\to R-COO^{-}-{Cr}^{6+}$$

(8) $$R-O^{-}+{Cr}^{6+}\to R-O^{-}-{Cr}^{6+}$$

(9) $$\;R-COOH+{Cr}^{6+}\to R-COO^{-}-{Cr}^{6+}+H^{+}$$

(10) $${Cr}_{2}O_{7}^{2-}+H_{2}O\to 2HCrO_{4}^{-}$$

(11) $$3R-O-R+3HCrO_{4}^{-}+5H^{+}\to 3R-O+3R-{Cr}^{3+}+4H_{2}O$$

(12) $$3R-OH+HCrO_{4}^{-}+4H^{+}\to 3R-O+{Cr}^{3+}+4H_{2}O$$

Finally, the chemisorption also works on the principle of sharing and transferring electron pairs between biochar and Cr (VI), with Ni_7_P_3_ acting as a high-charge-mobility electron donor during the absorption process, which is marked as (1) ion exchange in Figure 13. Most of the Cr (VI) (more than 80%) will be reduced to Cr (III) with the assistance of redox action. Meanwhile, the formation of Cr (III)-based precipitation and complexes is due to the (2) complexation and coprecipitation (4) process, as shown in Figure 13.

### 2.8. Comparison of Cr (VI) Adsorption Performances of Adsorbents

The Cr (VI) adsorption efficiency of PCNi_3_ was compared to that of other recently reported biochars to evaluate its potential practical application, and the results are presented in Table 5. Notably, PCNi_3_ exhibits an excellent Cr (VI) adsorption capacity compared to the other adsorbents mentioned in previous studies, which is mainly attributed to its sophisticated porous structure, remarkable specific surface area, and abundant oxygen-containing functional groups. In addition, the doped nickel can generate more micropores during calcination, and the formation of Ni^0^ particles contributes to the reduction of Cr (VI), providing rich active sites on the surface of biochar. Therefore, chestnut shell-based porous biochar modified by H_3_PO_4_ activation and nickel doping can be considered a promising adsorbent for the removal of Cr (VI) from wastewater.

## 3. Experimental Section

The final chestnut shell-derived porous magnetic biochar (abbreviated as PCNi) was effectively obtained by a combination of H_3_PO_4_-assisted two-step activation and a nickel doping method. More details about the preparation, characterization, and experimental results analysis are provided in the Supplementary Materials.

## 4. Conclusions

In this work, a series of chestnut shell-based porous biochars were successfully prepared by the treatment of H_3_PO_4_ activation and nickel doping. The as-obtained modified biochars possess loose porous microstructures, and their net structured surfaces covered by a layer of reticulate cotton-like substances can promote the charge transfer rate and contribute positively to adsorption performance. Among the biochars, PCNi_3_ has unique porous characteristics and a unique pore-size distribution, a high specific surface area of 1775.94 m^2^·g^−1^, abundant surface functional groups, and surface active sites, endowing it with excellent adsorption performance. Under optimal experimental conditions (pH = 2.0, C_e_ = 50 mg L^−1^, t_contact_ = 1400 min, and T = 298 K), PCNi_3_ exhibited a superior experimental Cr (VI) absorption capacity as high as 143.51 mg g^−1^. The CR (VI) adsorption isotherm data for PCNi_3_ fit the Freundlich model, implying that it has multilayer, non-uniform adsorption characteristics. The pseudo-second-order kinetic model and the Elovich model are suitable for simulating the kinetic data of PCNi_3_ with respect to Cr (VI), which indicates that the adsorption process is characterized by chemisorption and non-homogeneous diffusion. Thermodynamic studies confirm that the adsorption of Cr (VI) by PCNi_3_ is a spontaneous and thermodynamically favorable reaction. Our work presents a feasible way to design economical and highly active chestnut shell-derived biochar adsorbents for the elimination of Cr (VI) from wastewater.

## Figures and Tables

**Table 1:** Fitting parameters of adsorption isotherm models.

Sample	Langmuir			Freundlich			Sips			
	K_L_	q_m_	R^2^	K_F_	n_F_	R^2^	q_s_	m	K_S_	R^2^
PC	0.045	121.09	0.904	31.24	4.52	0.957	195.15	2.59	0.007	0.969
PCNi_3_	0.390	166.89	0.890	87.01	8.54	0.971	235.90	4.23	0.154	0.993

**Table 2:** Fitting parameters of pseudo-first-order, pseudo-second-order, and Elovich kinetics models.

Sample	Pseudo-First Order			Pseudo-Second Order			Elovich		
	k_1_	q_1_	R^2^	k_2_	q_2_	R^2^	α	β	R^2^
PC	0.014	57.92	0.790	3.33 × 10^−4^	63.33	0.913	5.18	0.098	0.993
PCNi_3_	0.007	132.08	0.976	5.59 × 10^−5^	152.97	0.997	3.72	0.035	0.986

**Table 3:** Fitting parameters of the intra-particle diffusion kinetics model.

Sample	Intra-Particle Diffusion					
	k_1_	C_1_	k_2_	C_2_	k_3_	C_3_
PC	7.366	−6.018	3.421	47.11	1.193	98.58
PCNi_3_	2.765	11.44	1.161	23.46	0.738	40.74

**Table 4:** Thermodynamic parameters for Cr (VI) adsorption by PC and PCNi_3_.

Sample	ΔG (kJ·mol^−1^)					ΔH	ΔS
	298 K	303 K	308 K	313 K	318 K	(kJ·mol^−1^)	J·(mol·K)^−1^
PC	−2.365	−1.911	−2.006	−1.735	−1.628	−10.03	−26.41
PCNi_3_	−4.902	−4.255	−5.523	−4.980	−6.019	11.81	55.40

**Table 5:** Adsorption performance of PCNi_3_ and other biochars.

Adsorbent	q_max_ (mg·g^−1^)	pH	Dosage	C_0_ (mg·L^−1^)	T (°C)	t_contact_ (h)	Refs.
Chitosan microspheres	24.16	3.0	1 g·L^−1^	25	25	24	[54]
Lemon peel	41.65	2.0	0.2 g·L^−1^	50	25	2	[55]
Alternanthera Philoxeroides	42.47	2.0	0.05 g	100	25	24	[56]
Sulfate-reducing sludge	58.56	3.0	0.8 g	50	25	24	[57]
Camellia oleifera shells	64.49	3.0	1 g·L^−1^	25	25	48	[58]
Chestnut shells	143.51	2.0	0.02 g	50	25	24	This work

## Data Availability

Data are contained within the article and Supplementary Materials.

## Supplementary Materials

The following supporting information can be downloaded at: https://www.mdpi.com/article/10.3390/molecules29102220/s1, Table S1: The surface elemental compositions of biochar (PC and PCNi) by EDS; Table S2: The pore structure parameters (textural properties) of PC and PCNi_3_ by BET and DFT method.

## References

[1] Syed A., Zeyad M.T., Shahid M., Elgorban A.M., Alkhulaifi M.M., Ansari I.A. (2021). Heavy Metals Induced Modulations in Growth, Physiology, Cellular Viability, and Biofilm Formation of an Identified Bacterial Isolate. ACS Omega.

[2] Gao X., Han G., Liu J., Zhang S., Zeng J. (2023). Heavy Metal Accumulation and Source Apportionment in Urban River under Ecological Restoration: Relationships with Land Use and Risk Assessment Based on Monte Carlo Simulation. ACS Earth Space Chem..

[3] Xia S., Liang S., Qin Y., Chen W., Xue B., Zhang B., Xu G. (2023). Significant Improvement of Adsorption for Phosphate Removal by Lanthanum-Loaded Biochar. ACS Omega.

[4] Xue S., Fan J., Wan K., Wang G., Xiao Y., Bo W., Gao M., Miao Z. (2021). Calcium-Modified Fe_3_O_4_ Nanoparticles Encapsulated in Humic Acid for the Efficient Removal of Heavy Metals from Wastewater. Langmuir.

[5] Rafieyan S.G., Marahel F., Ghaedi M., Maleki A. (2022). Application of Terminalia catappa wood-based activated carbon modified with CuO nanostructures coupled with H_2_O_2_ for the elimination of chemical oxygen demand in the gas refinery. J. Nanostruct. Chem..

[6] Cui P., Liu C., Su X., Yang Q., Ge L., Huang M., Dang F., Wu T., Wang Y. (2022). Atomically Dispersed Manganese on Biochar Derived from a Hyperaccumulator for Photocatalysis in Organic Pollution Remediation. Environ. Sci. Technol..

[7] Merino D., Zych A., Athanassiou A. (2022). Biodegradable and Biobased Mulch Films: Highly Stretchable PLA Composites with Different Industrial Vegetable Waste. ACS Appl. Mater. Interfaces.

[8] Khan Z.H., Li Z., Gao M., Islam S., Xiao L., Qiu W., Song Z. (2023). Simultaneous and efficient removal of Cd(II) and As(III) by a magnesium-manganese codoped biochar composite: Sorption performance and governing mechanisms. J. Environ. Chem. Eng..

[9] Zhang Y., Tang Y., Yan R., Li J., Li C., Liang S. (2023). Removal performance and mechanisms of aqueous Cr (VI) by biochar derived from waste hazelnut shell. Environ. Sci. Pollut. Res..

[10] Grimm A., Chen F., dos Reis G.S., Dinh V.M., Khokarale S.G., Finell M., Mikkola J.-P., Hultberg M., Dotto G.L., Xiong S. (2023). Cellulose Fiber Rejects as Raw Material for Integrated Production of *Pleurotus* spp. Mushrooms and Activated Biochar for Removal of Emerging Pollutants from Aqueous Media. ACS Omega.

[11] Zhao L., Li Q., Wang H., Zhou Z., Li N., Pan H., Liu Y., Liu X. (2023). Enhanced Adsorptive Removal of Tetracycline by Phosphomolybdic Acid-Modified Low-Temperature Sludge Biochar. Langmuir.

[12] Liu H., Cheng C., Wu H. (2021). Sustainable utilization of wetland biomass for activated carbon production: A review on recent advances in modification and activation methods. Sci. Total Environ..

[13] Feng P., Li J., Wang H., Xu Z. (2020). Biomass-Based Activated Carbon and Activators: Preparation of Activated Carbon from Corncob by Chemical Activation with Biomass Pyrolysis Liquids. ACS Omega.

[14] Zeng H., Zeng H., Zhang H., Shahab A., Zhang K., Lu Y., Nabi I., Naseem F., Ullah H. (2021). Efficient adsorption of Cr (VI) from aqueous environments by phosphoric acid activated eucalyptus biochar. J. Clean. Prod..

[15] Yang Z., Gleisner R., Mann D.H., Xu J., Jiang J., Zhu J.Y. (2020). Lignin Based Activated Carbon Using H_3_PO_4_ Activation. Polymers.

[16] Chen G., Wang H., Han L., Yang N., Hu B., Qiu M., Zhong X. (2021). Highly efficient removal of U(VI) by a novel biochar supported with FeS nanoparticles and chitosan composites. J. Mol. Liq..

[17] Yap M.W., Mubarak N.M., Sahu J.N., Abdullah E.C. (2017). Microwave induced synthesis of magnetic biochar from agricultural biomass for removal of lead and cadmium from wastewater. J. Ind. Eng. Chem..

[18] Jiang C., Zhou S., Li C., Yue F., Zheng L. (2022). Properties and mechanism of Cr(VI) removal by a ZnCl2-modified sugarcane bagasse biochar–supported nanoscale iron sulfide composite. Environ. Sci. Pollut. Res..

[19] Shen Y. (2015). Carbothermal synthesis of metal-functionalized nanostructures for energy and environmental applications. J. Mater. Chem. A.

[20] Li H., Dong X., da Silva E.B., de Oliveira L.M., Chen Y., Ma L.Q. (2017). Mechanisms of metal sorption by biochars: Biochar characteristics and modifications. Chemosphere.

[21] Lian F., Xing B. (2017). Black Carbon (Biochar) In Water/Soil Environments: Molecular Structure, Sorption, Stability, and Potential Risk. Environ. Sci. Technol..

[22] Pan Y., Li N., Qin X., Liu X., Shi S., Yan M., Liu Q., Liu Z. (2023). Effect of Microstructure and Macro Size of Walnut Shell Chars on Their Electromagnetic Induction Heating Behavior. Ind. Eng. Chem. Res..

[23] Wang K., Sun Y., Tang J., He J., Sun H. (2020). Aqueous Cr(VI) removal by a novel ball milled Fe0-biochar composite: Role of biochar electron transfer capacity under high pyrolysis temperature. Chemosphere.

[24] Xu J., Liu J., Ling P., Zhang X., Xu K., He L., Wang Y., Su S., Hu S., Xiang J. (2020). Raman spectroscopy of biochar from the pyrolysis of three typical Chinese biomasses: A novel method for rapidly evaluating the biochar property. Energy.

[25] Chen M., He F., Hu D., Bao C., Huang Q. (2020). Broadened operating pH range for adsorption/reduction of aqueous Cr(VI) using biochar from directly treated jute (*Corchorus capsularis* L.) fibers by H_3_PO_4_. Chem. Eng. J..

[26] Yasdi Y., Rinaldi R., Anggraini F.J., Yulianti T. (2021). Biochar from Oil Palm Frond to Reduce Fe Ions in Artificial Solution and Peat Water. Adv. Mater. Res..

[27] Deng Z., Deng Q., Wang L., Xiang P., Lin J., Murugadoss V., Song G. (2021). Modifying coconut shell activated carbon for improved purification of benzene from volatile organic waste gas. Adv. Compos. Hybrid Mater..

[28] Zeng B., Xu W., Khan S.B., Wang Y., Zhang J., Yang J., Su X., Lin Z. (2021). Preparation of sludge biochar rich in carboxyl/hydroxyl groups by quenching process and its excellent adsorption performance for Cr(VI). Chemosphere.

[29] Harvey O.R., Herbert B.E., Rhue R.D., Kuo L.-J. (2011). Metal Interactions at the Biochar-Water Interface: Energetics and Structure-Sorption Relationships Elucidated by Flow Adsorption Microcalorimetry. Environ. Sci. Technol..

[30] Ronsse F., van Hecke S., Dickinson D., Prins W. (2013). Production and characterization of slow pyrolysis biochar: Influence of feedstock type and pyrolysis conditions. GCB Bioenergy.

[31] Zhang N., Li J., Tian B., Li T., Zhang J., Zhao H. (2023). The preparation of amino-reinforced phosphorylated biochar for efficient uranium adsorption. J. Radioanal. Nucl. Chem..

[32] Ma L., Du Y., Chen S., Du D., Ye H., Zhang T.C. (2022). Highly efficient removal of Cr(VI) from aqueous solution by pinecone biochar supported nanoscale zero-valent iron coupling with Shewanella oneidensis MR-1. Chemosphere.

[33] Liu C., Wang W., Wu R., Liu Y., Lin X., Kan H., Zheng Y. (2020). Preparation of Acid- and Alkali-Modified Biochar for Removal of Methylene Blue Pigment. ACS Omega.

[34] Lyu H., Tang J., Huang Y., Gai L., Zeng E.Y., Liber K., Gong Y. (2017). Removal of hexavalent chromium from aqueous solutions by a novel biochar supported nanoscale iron sulfide composite. Chem. Eng. J..

[35] Yang H., Hong M., Chen S. (2020). Removal of Cr(VI) with nano-FeS and CMC-FeS and transport properties in porous media. Environ. Technol..

[36] He X., Min X., Peng T., Zhao F., Ke Y., Wang Y., Jiang G., Xu Q., Wang J. (2020). Mechanochemically Activated Microsized Zero-Valent Iron/Pyrite Composite for Effective Hexavalent Chromium Sequestration in Aqueous Solution. J. Chem. Eng. Data.

[37] Yang Y., Zhang Y., Wang G., Yang Z., Xian J., Yang Y., Li T., Pu Y., Jia Y., Li Y. (2021). Adsorption and reduction of Cr(VI) by a novel nanoscale FeS/chitosan/biochar composite from aqueous solution. J. Environ. Chem. Eng..

[38] Yi Y., Wang X., Ma J., Ning P. (2021). Fe(III) modified Egeria najas driven-biochar for highly improved reduction and adsorption performance of Cr(VI). Powder Technol..

[39] Ma R., Yan X., Pu X., Fu X., Bai L., Du Y., Cheng M., Qian J. (2021). An exploratory study on the aqueous Cr(VI) removal by the sulfate reducing sludge-based biochar. Sep. Purif. Technol..

[40] Liu Y., Ke X., Wu X., Ke C., Chen R., Chen X., Zheng X., Jin Y., Van der Bruggen B. (2020). Simultaneous Removal of Trivalent Chromium and Hexavalent Chromium from Soil Using a Modified Bipolar Membrane Electrodialysis System. Environ. Sci. Technol..

[41] Zhu Y., Guo C., Guan Q., He L., Zhou H., Xin H., He M., Zhang X., Liu R. (2024). Efficient Anchoring of Cu(II)–Tetracycline Complex in Paper Mill Sludge Biochar-Limited Nanospace. ACS EST Water.

[42] Peng X., Luo Z., Xie H., Liang W., Luo J., Dang C., Wang A., Hu L., Yu X., Cai W. (2022). Removal of phenylarsonic acid compounds by porous nitrogen doped carbon: Experimental and DFT study. Appl. Surf. Sci..

[43] Nguyen L.H., Van H.T., Nguyen Q.T., Nguyen T.H., Nguyen T.B.L., Nguyen V.Q., Bui T.U., Le Sy H. (2021). Paper waste sludge derived-hydrochar modified by iron (III) chloride for effective removal of Cr(VI) from aqueous solution: Kinetic and isotherm studies. J. Water Process Eng..

[44] Yao Y., Mi N., He C., He H., Zhang Y., Zhang Y., Yin L., Li J., Yang S., Li S. (2020). Humic acid modified nano-ferrous sulfide enhances the removal efficiency of Cr(VI). Sep. Purif. Technol..

[45] Zhu S., Ho S.-H., Huang X., Wang D., Yang F., Wang L., Wang C., Cao X., Ma F. (2017). Magnetic Nanoscale Zerovalent Iron Assisted Biochar: Interfacial Chemical Behaviors and Heavy Metals Remediation Performance. ACS Sustain. Chem. Eng..

[46] Zhang X. (2023). Study on the Adsorption Properties of Cr(VI) by Biochar with Different Treatments. Nat. Environ. Pollut. Technol..

[47] Liu N., Zhang Y., Xu C., Liu P., Lv J., Liu Y., Wang Q. (2020). Removal mechanisms of aqueous Cr(VI) using apple wood biochar: A spectroscopic study. J. Hazard. Mater..

[48] Zhu D., Shao J., Li Z., Yang H., Zhang S., Chen H. (2021). Nano nickel embedded in N-doped CNTs-supported porous biochar for adsorption-reduction of hexavalent chromium. J. Hazard. Mater..

[49] El-Kady A.A., Abdel-Wahhab M.A. (2018). Occurrence of trace metals in foodstuffs and their health impact. Trends Food Sci. Technol..

[50] Zaidi A., Wani P.A., Khan M.S. (2012). Toxicity of Heavy Metals to Legumes and Bioremediation.

[51] Suliman W., Harsh J.B., Abu-Lail N.I., Fortuna A.-M., Dallmeyer I., Garcia-Perez M. (2016). Influence of feedstock source and pyrolysis temperature on biochar bulk and surface properties. Biomass Bioenergy.

[52] Qu J., Wang Y., Tian X., Jiang Z., Deng F., Tao Y., Jiang Q., Wang L., Zhang Y. (2021). KOH-activated porous biochar with high specific surface area for adsorptive removal of chromium (VI) and naphthalene from water: Affecting factors, mechanisms and reusability exploration. J. Hazard. Mater..

[53] Feng Z., Chen N., Feng C., Gao Y. (2018). Mechanisms of Cr(VI) removal by FeCl3-modified lotus stem-based biochar (FeCl3@LS-BC) using mass-balance and functional group expressions. Colloids Surf. A Physicochem. Eng. Asp..

[54] Liu Y., Shan H., Pang Y., Zhan H., Zeng C. (2023). Iron modified chitosan/coconut shell activated carbon composite beads for Cr(VI) removal from aqueous solution. Int. J. Biol. Macromol..

[55] Ahmadian A., Goharrizi B.A., Shahriari T., Ahmadi S. (2023). Adsorption of chromium (VI) and Acid Orange 7 on lemon peel biochar: A response surface methodology approach. Int. J. Environ. Sci. Technol..

[56] Luo X., Du H., Zhang X., Yang Y. (2022). Amine-functionalized magnetic biochars derived from invasive plants Alternanthera philoxeroides for enhanced efficient removal of Cr(VI): Performance, kinetics and mechanism studies. Environ. Sci. Pollut. Res..

[57] Chen Y., Ma R., Pu X., Fu X., Ju X., Arif M., Yan X., Qian J., Liu Y. (2022). The characterization of a novel magnetic biochar derived from sulfate-reducing sludge and its application for aqueous Cr(VI) removal through synergistic effects of adsorption and chemical reduction. Chemosphere.

[58] Liu Y., Shan H., Zeng C., Zhan H., Pang Y. (2022). Removal of Cr(VI) from Wastewater Using Graphene Oxide Chitosan Microspheres Modified with α–FeO(OH). Materials.

