# Supplementary material for: Enhancing Cr (VI) Adsorption of Chestnut Shell Biochar through H3PO4 Activation and Nickel Doping
Source: Molecules. 2024 May 9;29(10):2220. doi: 10.3390/molecules29102220 (PMC11124291; doi:10.3390/molecules29102220)
Supplement: Supplementary file 1 [file molecules-29-02220-s001.zip › molecules-2978710-supplementary.pdf]

Supplementary Material for

Enhancing Cr (VI) adsorption of chestnut-shell biochar through H_3PO_4 activation and nickel doping

Hu Wen[1#], Zhang Xiaojing[2#], Chen Ming[2], Rahman Sheikh Tamjidur[2], Li Xin[2], Wang Geming[2, 3]*

(1. School of Information Engineering, Hubei University of Economics, Wuhan 430205, China; 2. Provincial Key Laboratory of Plasma Chemistry and Advanced Materials, Wuhan Institute of Technology, Wuhan 430073, China; 3. Hubei Key Laboratory of Industrial Fume and Dust Pollution Control, Jianghan University, Wuhan 430056, China)

Corresponding authors

E-mail address: wanggemingwit@163.com (G. Wang)

Tel: +86-027-87195661; Fax: +86-027-87195661

[#] Hu Wen and Zhang Xiaojing contributed equally to this work.

1. Experimental

1.1 Chemicals

Chestnut shells (residue from the manufacturer) were obtained from Wuhan, Hubei Province, China. All chemicals in our experiments were analytical reagent grade and used without further purification. Potassium dichromate ($K_2Cr_2O_7$), phosphoric acid (H_3PO_4), hydrochloric acid (HCl), sulfuric acid (H_2SO_4), sodium hydroxide (NaOH) and nickel chloride hexahydrate ($NiCl_2 \cdot 6H_2O$) were obtained from Sinopharm Chemical Reagent Co., Ltd., China. Deionized water was used for all experiments.

1.2 Biochar preparation

The original chestnut shells were washed, dried, ground and crushed, and then passed through a 50-mesh sieve. The chestnut shell-based porous biochar was synthesized by two-step H_3PO_4-assistant activation method. The treated chestnut shells were heated to 600°C at 5 °C/min in a tubular furnace under N_2 atmosphere and held for 2 h. The resulting products were washed, filtered and dried and passed through a 100-mesh sieve, which were recorded as BC. The sieved BC and H_3PO_4 were mixed in a mass ratio of 1:2 (BC: H_3PO_4=1:2) and dissolved in an appropriate amount of deionized water, and then the mixture was ultrasonically vibrated for 1 h, magnetically stirred for 6 h and dried. Subsequently, the mixture was heated to 950 °C at a rate of 5 °C/min under N_2 atmosphere and maintained for 3 h. The resulting products were washed and dried to obtain chestnut shell-based porous biochar, which was named as PC. The as-prepared PC and $NiCl_2 \cdot 6H_2O$ were dissolved in an appropriate amount of deionized water at different mass ratios (PC: $NiCl_2 \cdot 6H_2O$ = 3:1, 1:1, 1:3), and magnetically stirred for 12h after ultrasonication for 1 h. After drying, the mixtures were calcined at 800 °C for 1 h under N_2 atmosphere, and stored for later use after natural cooling, washing and drying. The final chestnut shell-based porous biochar (abbreviate as PCNi) was recorded as $PCNi_1$, $PCNi_2$ and $PCNi_3$, respectively. The $PCNi_3$ after the absorption process of Cr (VI) was recorded as $PCNi_3$-Cr.

1.3 Biochar characterization

The crystalline structures of samples were acquired by X-ray powder diffraction (XRD, Germany Brucker D8 ADVANCE) using Cu Kα radiation (λ = 1.5406 Å, 2θ

=10°-80°). The surface feature, microstructure and chemical compositions of PC, PCNi and $PCNi_3$-Cr were investigated by a field emission scanning electron microscope (FE-SEM, ZEISS GeminiSEM 300) equipped with an energy-dispersive spectroscopy (EDS) mapping instrument was employed to determine surface element contents. Raman spectra (Raman, Thermo Fisher DXR) of BC, PC and $PCNi_3$ were obtained to analyze the variation of carbon defect and graphitization degree. The surface chemical functional groups of BC, PC and $PCNi_3$ are characterized by using a Nicolet 6700 Fourier transform-infrared (FT-IR) spectrophotometer. The pore volume, pore-size distribution and the Brunauer-Emmett-Teller (BET) specific surfaces areas of BC, PC and $PCNi_3$ were measured by a BET specific surface area analyzer (Mike ASAP2020) following the multipoint N_2 adsorption/desorption BET method. The related data are calculated from the N_2 adsorption isotherms by using the Brunauer-Emmett-Teller (BET) and density functional theory (DFT) method. X-ray photoelectron spectroscopy (XPS, Thermo Fisher ESCALAB XI+) was carried out to explore the surface chemical compositions and elemental valence states of PC, $PCNi_3$ and $PCNi_3$-Cr. The room-temperature magnetic properties of $PCNi_3$ were investigated with vibrating sample magnetometer (VSM, Lakeshore 7404).

1.4 Adsorption experiments

All adsorption experiments of as-prepared biochar were conducted in 150 ml conical flasks containing 100 ml of Cr (VI) solution with biochar dosage of 20 mg. In order to ensure adequate adsorption, the conical flasks were shaken in a thermostat oscillator with 150 rpm. Batch experiments were carried out to reveal adsorption behavior and mechanism *via* initial solution pH, initial Cr (VI) solution concentration, reaction time and temperature. The influence of pH on Cr (VI) solution toward biochar were recorded by dropping 0.1 mol/ L H_2SO_4 or 0.1 mol/L NaOH to adjust initial pH value of the reaction solution from 2.0 to 8.0 while maintaining Cr (VI) concentration at 50 mg L^{-1} for 1440 min. The suitable solution pH was determined as a fixed value (pH 2.0) for the following adsorption experiments. The impacts of key changes include initial Cr (VI) solution concentration (5, 10, 25, 50, 100, 200, 300, 400, 500 and 600 mg L^{-1}), reaction time (10, 20, 30, 60, 90, 120, 180, 360, 540, 720, and 1440 min) and

temperature (298, 303, 308, 318 and 323K) on adoption capacity toward Cr (VI) have been studied by adsorption experiments. The reaction solution was filtered through a 0.22 μm syringe filter and collected before the final concentration was tested. Furthermore, the pseudo-first order kinetic, pseudo-second order kinetics kinetic, Elovich and intra-particle diffusion were used to fit experiment data, and the thermodynamic parameters including entropy (ΔS^0, J mol^{-1} K^{-1}), enthalpy (ΔH^0, KJ mol^{-1}) and Gibbs free energy (ΔG^0, KJ mol^{-1}) were also calculated.

1.5 Data analysis

1.5.1 Adsorption capacity

The concentration of Cr (VI) was measured by diphenylcarbazide colorimetry with a UV-Vis spectrophotometer at 540 nm. The adsorption capacity (q_e, mg·g^{-1}) were calculated by Eqs. (S1) and (S2):

$$\eta(\%) = \frac{C_o - C_e}{C_o} \times 100\% \quad \text{(S1)}$$

$$q_e = \frac{(C_o - C_e)V}{m} \quad \text{(S2)}$$

Where, C_o (mg·L^{-1}) and C_e (mg·L^{-1}) are the initial concentration and equilibrium concentrations, V (L) is the solution volume, and m (g) is the mass of biochar.

1.5.2 Kinetic model

According to Eqs. (S3) ~ (S6), the pseudo-first-order, pseudo-second, Intra-particle diffusion models and Elovich were used in turn to model the kinetics of Cr (VI) adsorption:

$$q_t = q_e(1 - e^{-k_1 t}) \quad \text{(S3)}$$

$$q_t = \frac{k_2 t q_e^2}{1 + k_2 t q_e} \quad \text{(S4)}$$

$$q_t = k_p t^{1/2} + C_i \quad \text{(S5)}$$

$$q_t = \frac{1}{\beta}\ln(\alpha\beta) + \frac{1}{\beta}\ln t \quad \text{(S6)}$$

In which, q_t (mg·g^{-1}) represent adsorption capacity at time t; k_1 (min^{-1}), k_2 (g mg^{-1} min^{-1}) and k_p (g mg^{-1} min^{-1}) are the corresponding rate constants for each model; α (mg g^{-1} min^{-1}) and β (mg·g^{-1}) are the initial adsorption rate and Elovich desorption constants; C_i is a constant and it represent the intercept of intra-particle diffusion plot.

1.5.2 Adsorption isotherms

According to Eqs. (S7) ~ (S9), three well-known isotherm models of Langmuir model considering a monolayer adsorption, Freundlich model considering a multilayer adsorption and Sips model considering the interaction force between the type of sorbent and adsorbent were carried out to study and predict the adsorption performance at equilibrium:

$$q_e = \frac{q_m K_L C_e}{1+K_L C_e} \tag{S7}$$

$$q_e = K_F C_e^{1/n_F} \tag{S8}$$

$$q_e = q_s (K_S C_e)^{1/m} / [(1 + (K_S C_e)^{1/m})] \tag{S9}$$

In which, q_m ($mg \cdot g^{-1}$) represent the Langmuir maximum adsorption capacity; q_e ($mg\ g^{-1}$) is the adsorption capacity at equilibrium; C_e ($mg\ L^{-1}$) mean the equilibrium concentration; K_L ($L\ mg^{-1}$) relate to the constant of Langmuir; K_F [($mg\ g^{-1}$) ($mg\ L^{-1})^{-n}$] and n_F is the constant of Freundlich; K_S ($L\ mg^{-1}$) and m are parameters of Sips adsorption model.

1.5.4 Thermodynamics studies

The corresponding thermodynamic parameters of entropy (ΔS^0, $KJ\ mol^{-1}\ K^{-1}$), enthalpy (ΔH^0, $KJ\ mol^{-1}$) and Gibbs free energy (ΔG^0, $KJ\ mol^{-1}$) were calculated and determined based on Eqs. (S10) ~ (S12).

$$\Delta G = -RT \ln K_d \tag{S10}$$

$$K_d = \frac{q_e}{C_e} \tag{S11}$$

$$\ln K_d = \frac{\Delta S}{R} - \frac{\Delta H}{RT} \tag{S12}$$

Where, T (K) is the temperature, R ($J\ mol^{-1}\ K^{-1}$) is the gas constant (~8.314), and K_d ($L\ g^{-1}$) means the equilibrium constant.

2. Results

Table S1 the surface elemental compositions of biochar (PC and PCNi) by EDS

sample	Elemental Compositions (%)			
	C	O	P	Ni
PC	92.43	7.30	0.27	—
PC_3Ni_1	86.50	8.71	3.01	1.79
PC_1Ni_1	65.26	18.14	5.72	8.77
PC_1Ni_3	64.41	21.68	4.07	8.31

Table S2 the pore structure parameters (textural properties) of PC and $PCNi_3$ by BET and DFT method

Pore structure parameters	PC	$PCNi_3$
specific surface area ($m^2 \cdot g^{-1}$)	1521.55	1775.94
microporous area ($m^2 \cdot g^{-1}$)	1076.77	1235.85
total pore volume ($cm^{-3} \cdot g^{-1}$)	0.80	0.97
micro pore volume ($cm^{-3} \cdot g^{-1}$)	0.63	0.73
average pore size (nm)	2.11	2.18
